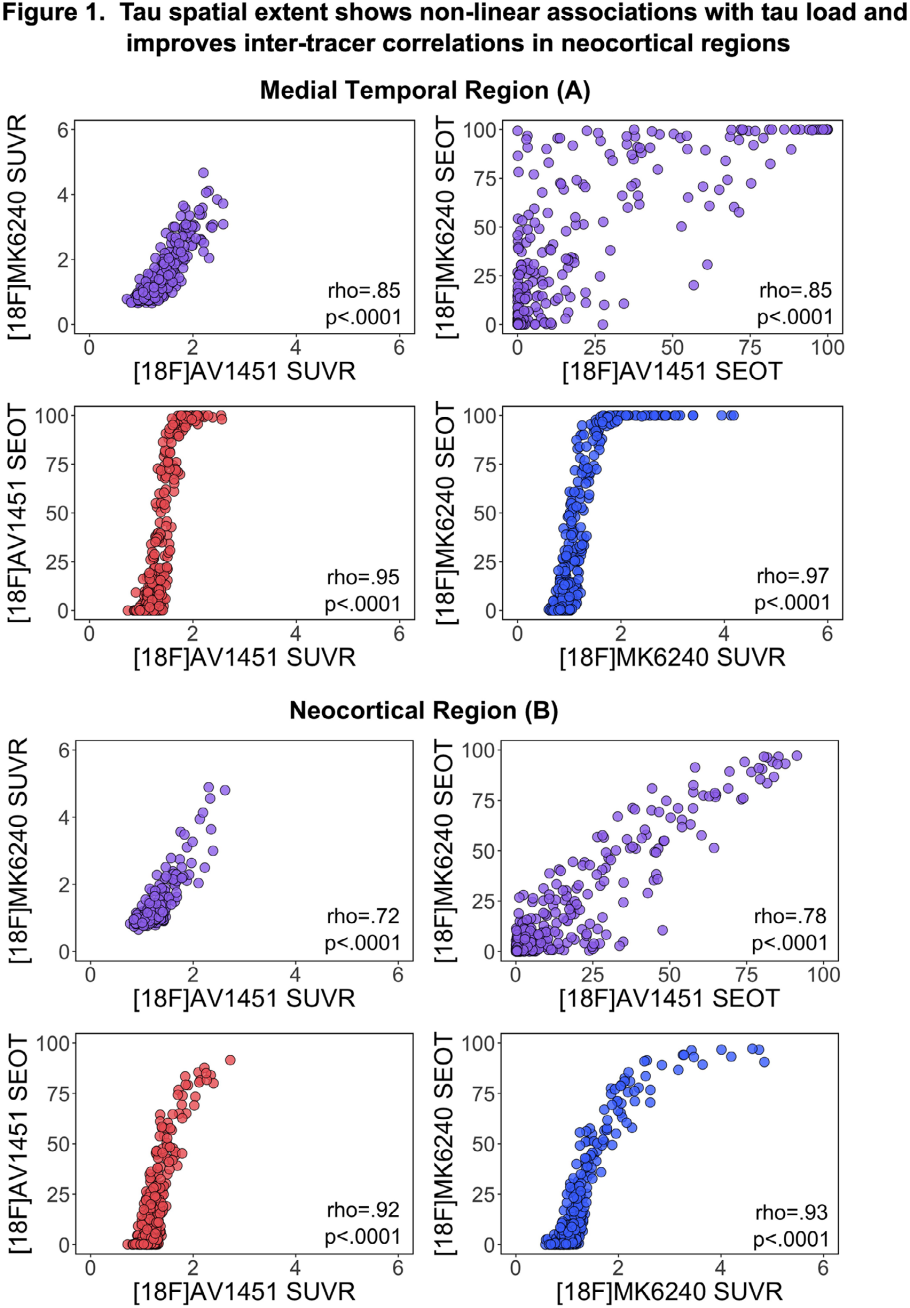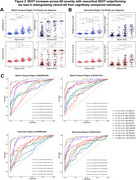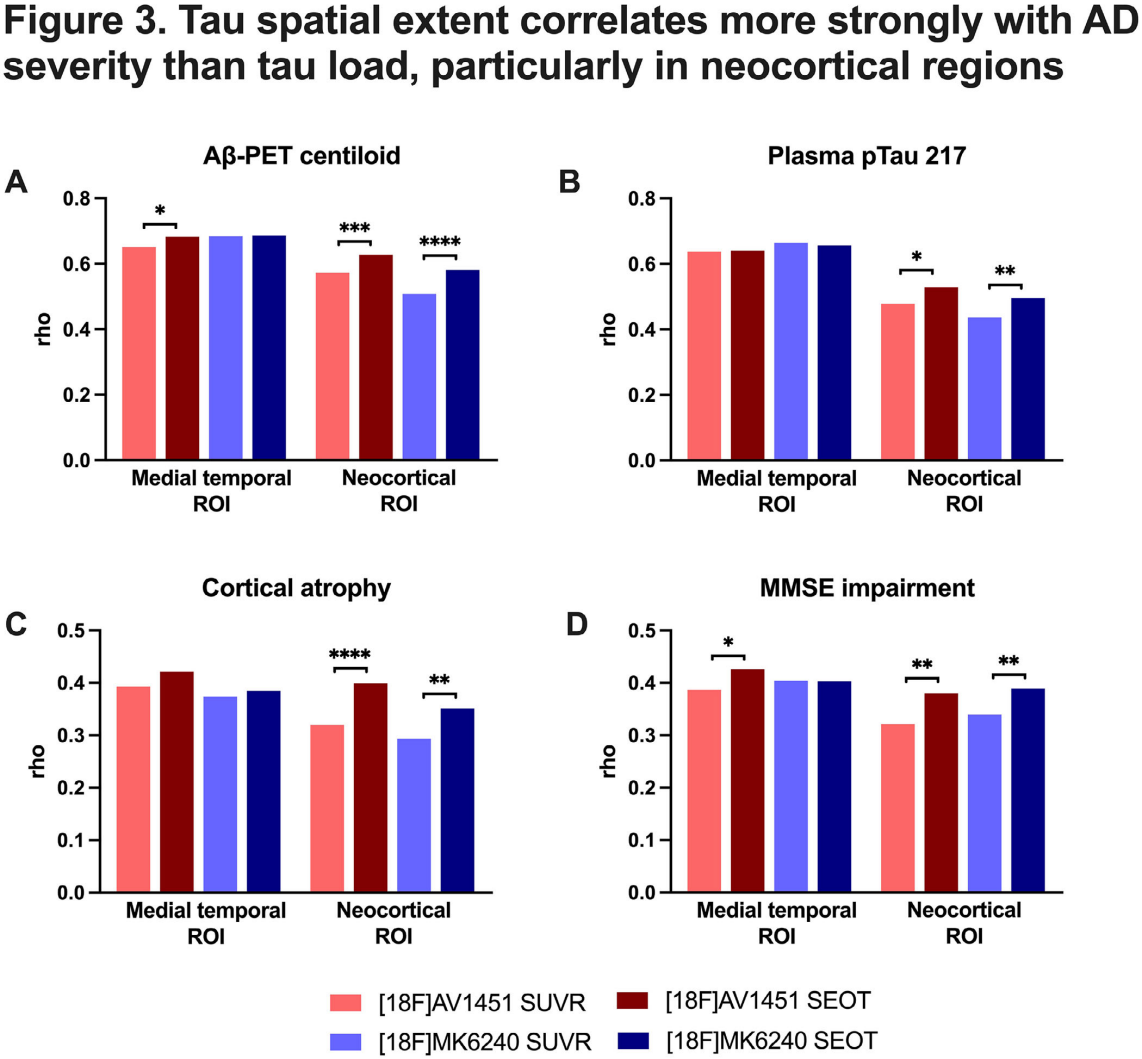# Tau Spatial Extent Outperforms Tau Load as a Marker of Neocortical Tau Burden: Head‐to‐head evidence from [18F]MK6240 and [18F]FTP PET

**DOI:** 10.1002/alz70856_105999

**Published:** 2026-01-08

**Authors:** Arthur C. Macedo, Nesrine Rahmouni, Firoza Z Lussier, Cécile Tissot, Gleb Bezgin, Joseph Therriault, Stijn Servaes, Seyyed Ali Hosseini, Brandon J Hall, Tevy Chan, Etienne Aumont, Pamela C.L. Ferreira, Bruna Bellaver, Guilherme Povala, Guilherme Bauer‐Negrini, Livia Amaral, Belen Pascual, Brian A. Gordon, Val J Lowe, Hwamee Oh, David N. Soleimani‐Meigooni, Juan Fortea, Suzanne L. Baker, Tharick A Pascoal, Pedro Rosa‐Neto

**Affiliations:** ^1^ McGill University, Montreal, QC, Canada; ^2^ Lawrence Berkeley National Laboratory, Berkeley, CA, USA; ^3^ Université du Québec à Montréal, Montréal, QC, Canada; ^4^ University of Pittsburgh, Pittsburgh, PA, USA; ^5^ Houston Methodist Neurological Institute, Houston, TX, USA; ^6^ Washington University in St. Louis, School of Medicine, St. Louis, MO, USA; ^7^ Mayo Clinic, Rochester, MN, USA; ^8^ Brown University, Providence, RI, USA; ^9^ University of California, San Francisco, San Francisco, CA, USA; ^10^ Sant Pau Memory Unit, Hospital de la Santa Creu i Sant Pau, Biomedical Research Institute Sant Pau, Barcelona, Spain

## Abstract

**Background:**

The 2024 NIA‐AA criteria propose an Alzheimer's disease (AD) staging system based on medial temporal (MT) and neocortical tau burden. However, the optimal strategy to assess tau burden in these regions, which represent distinct phases of AD pathophysiology, remains unclear. Here, we compared tau load and spatial extent of tauopathy (SEOT) as markers of tau burden in participants scanned with [^18^F]MK6240 and [^18^F]FTP. We investigated the relationship between these metrics in MT and neocortical regions‐of‐interest (ROIs) and determined which better correlates with AD severity.

**Method:**

A total of 257 cognitively unimpaired (CU) and 124 Aβ+ cognitively impaired participants (mean age 68.2 years; 58% female) from the HEAD study underwent tau‐PET imaging with both [^18^F]MK6240 and [^18^F]FTP. Tau load was quantified using regional SUVR, and SEOT was calculated as the proportion of abnormal voxels relative to young controls. Both metrics were derived from the MT and neocortical ROIs. Spearman's correlations were computed between SUVR and SEOT within the same tracer and across tracers in the same ROIs. We compared rho coefficients to assess whether one metric showed stronger concordance. Additionally, we compared the correlations of SEOT and SUVR with global cognition, cortical thickness, Aβ‐PET load, and plasma pTau‐217. Statistical comparisons were performed using the R package *cocor*.

**Result:**

SUVR and SEOT exhibited non‐linear correlations in both MT and neocortical ROIs for both tracers, with SEOT enhancing the correlation between [^18^F]MK6240 and [^18^F]FTP in the neocortex (*p* <.0001; Figure 1). Both metrics increased with AD severity, with MT SUVR outperforming SEOT in distinguishing Aβ‐ CU from Aβ+ CU individuals, and neocortical SEOT outperforming SUVR in distinguishing Aβ+ MCI from CU (Figure 2). Neocortical SEOT showed stronger correlations with AD severity markers than SUVR for both tracers. MT SEOT was comparable to SUVR, outperforming SUVR only in correlations with MMSE and Aβ‐PET for [^18^F]FTP (Figure 3).

**Conclusion:**

Tau extent improves the association of tau‐PET tracers with AD severity, particularly in the neocortex. These findings suggest that tau extent might be a more suitable surrogate marker of tau burden in neocortical regions, offering valuable insights for in vivo staging of tau pathology and clinical trials targeting late‐stage tau pathology.